# Preventive sparing of spinal cord and brain stem in the initial irradiation of locally advanced head and neck cancers

**DOI:** 10.1120/jacmp.v15i1.4399

**Published:** 2014-01-06

**Authors:** Paolo Farace, Sara Piras, Sergio Porru, Federica Massazza, Giuseppina Fadda, Ignazio Solla, Denise Piras, Maria Assunta Deidda, Maurizio Amichetti, Marco Possanzini

**Affiliations:** ^1^ Department of Radio‐Oncology Regional Oncological Hospital Cagliari Italy; ^2^ Protontherapy Unit APSS Trento Italy

**Keywords:** radiotherapy, IMRT, SIB, head and neck, spinal cord, brain stem, DMPO

## Abstract

Since reirradiation in recurrent head and neck patients is limited by previous treatment, a marked reduction of maximum doses to spinal cord and brain stem was investigated in the initial irradiation of stage III/IV head and neck cancers. Eighteen patients were planned by simultaneous integrated boost, prescribing 69.3 Gy to PTV1 and 56.1 Gy to PTV2. Nine 6 MV coplanar photon beams at equispaced gantry angles were chosen for each patient. Step‐and‐shoot IMRT was calculated by direct machine parameter optimization, with the maximum number of segments limited to 80. In the standard plan, optimization considered organs at risk (OAR), dose conformity, maximum dose <45 Gy to spinal cord and <50 Gy to brain stem. In the sparing plans, a marked reduction to spinal cord and brain stem were investigated, with/without changes in dose conformity. In the sparing plans, the maximum doses to spinal cord and brain stem were reduced from the initial values (43.5±2.2 Gy and 36.7±14.0 Gy), without significant changes on the other OARs. A marked difference (−15.9±1.9 Gy and −10.1±5.7 Gy) was obtained at the expense of a small difference (−1.3%±0.9%) from initial $\text{PTV}1_{95\%}$ coverage (96.6%±0.9%). Similar difference (−15.7±2.2 Gy and −10.2±6.1 Gy) was obtained compromising dose conformity, but unaffecting ${\text{PTV}}_{195\%}$ and with negligible decrease in $\text{PTV}2_{95\%}$ (−0.3%±0.3% from the initial 98.3%±0.8%). A marked spinal cord and brain stem preventive sparing was feasible at the expense of a decrease in dose conformity or slightly compromising target coverage. A sparing should be recommended in highly recurrent tumors, to make potential reirradiation safer.

PACS number: 87.55.D

## I. INTRODUCTION

Locoregional recurrences are common in head and neck cancer patients, both after adjuvant or definitive radiotherapy. While a large portion of patients presenting with stage I and II squamous cell carcinoma of the head and neck will remain disease free after single modality treatment, the patients presenting in a more advanced disease (stage III/IV) will eventually relapse.^(1)^ An overall five‐year survival rate of around 50% demonstrates that treatment is often unsuccessful.® Whereas better results can be obtained in oropharyngeal cancer with the use of modern irradiation techniques,^3^, ^4^ the outcome in carcinomas of the larynx and the hypopharynx using the most aggressive chemoradiation approaches remains poor.^5^, ^6^ Even after multimodal treatment local and locoregional recurrence remains the cause of poor survival statistics. Patients treated with chemoradiation can relapse with three classic determining factors of outcome after radiotherapy: intrinsic radiosensitivity, hypoxia, and repopulation rate.^2^, ^7^ Moreover, multiple retrospective analyses have indeed indicated that HPV‐associated tumors are more sensitive to chemoradiotherapy than nontransformed tumors, and the prognostic value of HPV status is now well‐established.^(8)^

Surgical salvage for resectable disease is the standard treatment for recurrent or second primary head and neck cancers, but it can be performed in 25% of the patients only. For inoperable patients, three options can be discussed: supportive care only, chemotherapy, or radiotherapy with or without chemotherapy.^(9)^ Definitive reirradiation is an established approach for patients with recurrent disease who are medically or technically inoperable or for patients who decline radical surgery due to quality‐of‐life considerations^(7)^ In addition, reirradiation is a candidate to become a more common practice in the patients who undergo salvage surgery. In fact, in these patients the rate of locoregional failure is as high as 50%,^(10)^ and even patients who undergo complete resection of recurrent disease with negative margins have a high risk of local failure.^(11)^ Accordingly, a randomized trial^(10)^ and a single‐institution experience^(12)^ found improved local control and overall survival in patients undergoing reirradiation for recurrent head and neck cancer. It has been argued that both chemo‐reirradiation after salvage surgery and close observation with delayed chemo‐reirradiation are justifiable^(13)^

Since recurrent and second primary tumors commonly arise within or in close proximity to previously irradiated fields, the radiation tolerance of normal tissue makes reirradiation technically challenging and frequently more toxic than the initial course.^(14)^ These patients have a poor prognosis to a large extent because the initial course of treatment substantially reduces the flexibility and the intensity of retreatment.^(15)^ One unexplored possibility is the preventive reduction, in the initial irradiation, of the dose to the organs at risk (OAR), which could limit the successive reirradiation. In fact, lifetime clinically acceptable dose is considered one of the main limiting factors in the reirradiation of recurrent patients.

In the recent reports using IMRT in the reirradiation setting,^15^, ^16^, ^17^, ^18^, ^19^, ^20^ the primary avoidance structures were spinal cord and brain stem. In these studies, the primary goals of inverse planning were to ensure homogeneous PTV coverage limiting the additional spinal cord/brain stem doses to 10–15 Gy or their lifetime doses to 55–60 Gy. Accordingly, radiobiological research indicates that the dose for 5% myelopathy is 59.3 Gy.^(21)^ Moreover, data in animals and humans suggest partial repair of radiation induced subclinical damage; when the interval between the two irradiation courses was as least two month, few cases of myelopathy were reported despite large cumulative doses, with essentially no cases of myelopathy observed for cumulative doses ≤60 Gy in 2 Gy equivalent doses (QUANTEC initiative^(22)^).

In the present study, we have investigated the relationships between a marked reduction of spinal cord and brain stem maximum doses and the corresponding changes on dose distributions. The consequences of such marked dose reduction were analyzed in the initial irradiation of 18 patients affected by locally advanced head and neck cancer planned by simultaneous integrated boost (SIB) intensity‐modulated radiotherapy (IMRT). In particular, our analysis focused on: i) the changes in target coverage, ii) the changes in dose conformity, and iii) the changes in the other OARs.

## II. MATERIALS AND METHODS

### A. Subjects and volume delineation

Eighteen consecutive patients with locally advanced (stage III/IV) head and neck cancer were investigated. Tumor localization and disease stage are reported in Table 1. All the patients were planned for definitive radiotherapy concurrent with chemotherapy.

**Table 1:** Tumor characteristics

*#*	*Site*	*Stage*	*PTV1 (cc)*	*PTV2 (cc)*
1	Oropharynx	T4a N2c	134	443
2	Larynx	T4a N2b	418	665
3	Hypopharynx	T2 N2c	306	635
4	Oral cavity	T4a N2	167	678
5	Oropharynx	T4a N1	295	481
6	Oropharynx	T3 N2c	147	468
7	Oropharynx	T3 N2	489	757
8	Oropharynx	T4a N2b	206	810
9	Oropharynx	T4a N1	433	795
10	Hypopharynx	T2 N2c	171	568
11	Oropharynx	T4 N0	340	761
12	Oropharynx	T4 N1	371	680
13	Oral cavity	T3 N3	206	498
14	Larynx	T3 N0	172	403
15	Oropharynx	T4a N1	220	471
16	Oropharynx	T4a N2c	271	669
17	Oral cavity	T3 N0	65	401
18	Rhinopharynx	T3 N3	438	1165

The gross tumor volume extent (GTV) has been identified on contrast‐enhanced CT. A high risk clinical target volume (CTV1), containing the GTVs plus a margin of at least 5 mm, was delineated to account for microscopic disease spread. A low risk CTV2 was delineated to include the prophylactically treated neck. The corresponding PTV1 and PTV2 were obtained expanding the CTVs by 5 mm, and then trimmed to 5 mm away from the patient's surface. Smaller PTVs ($\text{PTV}1_{3\text{mm}}$ and $\text{PTV}2_{3\text{mm}}$) were obtained using a 3 mm expansion from CTVs.

The following OARs were contoured on simulation CT: spinal cord, brain stem, parotids, mandible, thyroid, oral mucosa, constrictor muscles, and larynx. When the OARs were completely included in the PTVs (the larynx in patient #2 and #14), they were not contoured. A planning organ‐at‐risk volume (PRV) was defined for spinal cord and brain stem, expanding the corresponding OAR by both 3 mm and 5 mm.

### B. Standard treatment planning

All the patients were retrospectively planned by SIB IMRT, prescribing 69.3 Gy in 33 fractions to PTV1 and 56.1 Gy to PTV2. Treatment planning was performed by Pinnacle (release 8.0; Philips Medical System, Andover, MA). Nine 6 MV coplanar photon beams at equispaced gantry angles were chosen for each patient, as in the study by Wu et al.^(23)^ This improves the plan quality, considering that our multileaf collimator had a leaf width of 10 mm.^(24)^ Step and shoot IMRT was calculated by direct machine parameter optimization (DMPO), with 4 cm^2^ minimum segment areas and with segment weights limited to 4 MUs. The maximum number of segments was limited to 80, considering that other authors reported that, for more complex cases, a dosimetric gain can be achieved by increasing the number of apertures per beam direction, but only modest improvements were observed beyond nine apertures per beam direction.^(25)^

The initial objectives applied on PTVs and OARs during IMRT optimization are reported in Table 2, together with two additional objectives applied to control dose conformity (last two rows in Table 2). In the first calculation, the weights were set equal to 50 for PTVs, equal to 100 for spinal cord and brain stem, and equal to 1 for the other OARs. The calculation was then repeated four to five times, manually changing the weights and the doses of the objectives until a satisfactory dose distribution was obtained as a compromise between PTVs coverage and OARs sparing.

**Table 2:** Objectives applied during IMRT optimization. The objective values are reported in Gy; their relative weights are reported in brackets

		*Standard*	*Moderate‐Sparing*	*Marked‐Sparing*	*Low‐Conformit*
PTV1	uniform dose	=69.3 (50)	Optimized^a^	Optimized^a^	Optimized^a^
PTV2‐1^b^	uniform dose	=56.1 (50)	Optimized^a^	Optimized^a^	Optimized^a^
spinal cord	max‐dose	<45 (100)	<35 (100)	<26.5 (100)	<26.5 (100)
brain stem	max‐dose	<50 (100)	<40 (100)	<31.5 (100)	<31.5 (100)
${\text{PRV}}_{3\text{mm}}$ spinal cord	max‐dose	<50 (100)	<50 (100)	<50 (100)	<50 (100)
PRV3 brain stem	max‐dose	<54 (100)	<54 (100)	<54 (100)	<54 (100)
mandible	max‐dose	<70 (1)	Optimized^a^	Optimized^a^	Optimized^a^
left parotid	mean‐dose	<26 (1)	Optimized^a^	Optimized^a^	Optimized^a^
right parotid	mean‐dose	<26 (1)	Optimized^a^	Optimized^a^	Optimized^a^
Thyroid	mean‐dose	<40 (1)	Optimized^a^	Optimized^a^	Optimized^a^
oral mucosa	mean‐dose	<40 (1)	Optimized^a^	Optimized^a^	Optimized^a^
constrictor muscles	mean‐dose	<45 (1)	Optimized^a^	Optimized^a^	Optimized^a^
larynx	mean‐dose	<45 (1)	Optimized^a^	Optimized^a^	Optimized^a^
0.5 cm ring around PTV1	max‐dose	<65.8 (50)	<65.8 (50)	<65.8 (50)	<65.8 (1)
body tissue outside PTV2	max‐dose	<53.3 (50)	<53.3 (50)	<53.3 (50)	<53.3 (1)

a ^a^ Optimized are the values and weights obtained in the optimization of the standard plan, when the calculation was repeated 4‐5 times, manually changing the weights and the doses of the objectives. These values were no more changed in the other plans.

b ^b^ PTV2‐1 was defined as the region formed by PTV2 from which PTV1 was subtracted.

### C. Sparing treatment plans

After the standard plan was calculated, a second plan (moderate‐sparing plan) was obtained, changing only the objectives of spinal cord and of brain stem, to obtain a moderate sparing but leaving the other objectives/weights optimized in the standard plan unchanged (Table 2).

A third plan (marked‐sparing plan) was obtained by reducing the maximum dose objectives of spinal cord and of brain stem to 26.5 and 31.5 Gy, respectively (Table 2). These values were chosen considering that median cumulative maximum doses of 53 Gy to the spinal cord and 63 Gy to the brain stem did not produced late toxicity in a recent study.^(26)^ Limiting the dose in the initial irradiation to around half of these values would produce the same limitation to any potential reirradiation.

Finally, a fourth plan (low‐conformity plan) was obtained by allowing a decrease in dose conformity when sparing the dose to spinal cord and to brain stem. This was obtained by setting weight=1 to the two objectives applied to control dose conformity (Table 2).

### D. Plan evaluation

In each patient, the four treatment plans were compared by the following metrics: maximum doses to spinal cord, brain stem and to their PRVs, mean doses to parotids, mandible, thyroid, oral mucosa, constrictor muscles, larynx, $\text{PTV}1_{95\%}$ (percentage of PTV1 receiving at least 95% of its prescribed dose), and $\text{PTV}2_{95\%}$. The 95% coverage was also assessed on $\text{PTV}1_{3\text{mm}}$ and on $\text{PTV}2_{3\text{mm}}$. Two conformity indexes (CI) were defined as the ratio of PTV1 (${\text{CI}}_{\text{PTV}1}$) or PTV2 (${\text{CI}}_{\text{PTV}2}$) covered by 95% isodose line divided by total tissue volume covered by that isodose line. Finally, the total monitor units (MU) were compared to assess the eventual increase in treatment complexity.

For each parameter comparison, statistical significance was calculated by paired Wilcoxon test.

## III. RESULTS

**Figure 1:**
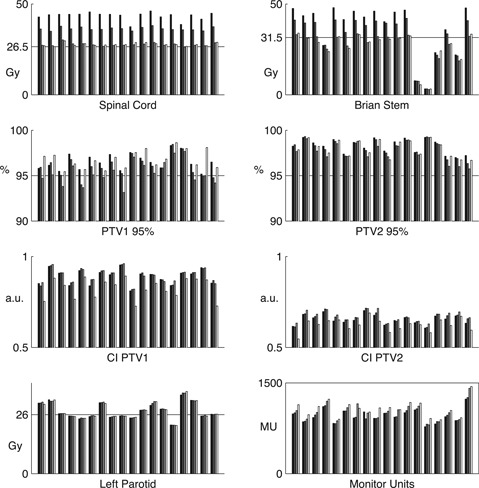
Bar graphs of the 18 patients; for each patient, a group of four bars is reported, which represent the four plans: standard plan (black bars), moderate‐sparing plan (dark gray bars), marked‐sparing plan (bright gray bars), and low‐conformity plan (white bars). Maximum doses are reported for spinal cord and brain stem, and mean‐dose are reported for the left parotid. The horizontal lines in the graph of spinal cord and brain stem represent the objective values utilized during optimization of marked‐sparing and low‐conformity plan, and in the other graphs they represent the recommended values. Since the OAR experienced no significant change across the four plans they were not reported. The left parotid is reported as an example to show interpatient variability.

In each inverse plan the number of segments resulted equal to the maximum allowable (80 segments). The comparison of the standard plan versus the three sparing plans is reported in Table 3 and Fig. 1.

**Table 3:** Standard versus sparing plans. Mean±standard deviation

			*Difference with Standard Plan*		
	*Units*	*Standard Plan*	*Moderate‐Sparing*	*Marked‐Sparing*	*Low‐Conformi*
Spinal Cord ($D_{\text{max}}$)	Gy	43.5±2.2	−7.1±1.8 ^a^	−15.8±2.0 ^a^	−15.7±2.2 ^a^
Brian Stem ($D_{\text{max}}$)	Gy	36.7±14.0	−3.7±2.7 ^a^	−10.0±5.7 ^a^	−10.2±6.0 ^a^
${\text{PRV}}_{3\text{mm}}$ Spinal Cord ($D_{\text{max}}$)	Gy	46.5±2.6	−5.3±1.7 ^a^	−13.3±2.7 ^a^	−12.3±2.6 ^a^
${\text{PRV}}_{3\text{mm}}$ Brian Stem ($D_{\text{max}}$)	Gy	39.6±13.9	−3.3±2.5 ^a^	−9.2±5.1 ^a^	−9.4±5.0 ^a^
${\text{PRV}}_{5\text{mm}}$ Spinal Cord($D_{\text{max}}$)	Gy	50.3±3.5	−3.9±2.0 ^a^	−10.7±3.1 ^a^	−9.4±2.5 ^a^
${\text{PRV}}_{5\text{mm}}$ Brian Stem ($D_{\text{max}}$)	Gy	42.8±13.6	−3.0±2.3 ^a^	−8.4±4.0 ^a^	−8.7±3.9 ^a^
$\text{PTV}1_{95\%}$	%	96.6±0.9	−0.5±0.6 ^a^	−1.2±0.8 ^a^	0.2±1.0
$\text{PTV}1_{(3\text{mm})95\%}$	%	99.3±0.9	−0.1±0.3	−0.3±0.5 ^a^	−0.1±0.4
$\text{PTV}2_{95\%}$	%	98.3±0.8	−0.2±0.2 ^a^	−0.6±0.4 ^a^	−0.3±0.3 ^a^
$\text{PTV}2_{(3\text{mm})95\%}$	%	99.5±0.4	−0.1±0.1 ^a^	−0.2±0.2 ^a^	−0.1±0.1 ^a^
Right Parotid ($D_{\text{mean}}$)	Gy	31.1±10.5	−0.0±0.2	0.1±0.4	0.3±0.6
Left Parotld ($D_{\text{mean}}$)	Gy	27.7±3.6	0.1±0.3	0.2±0.5	0.1±0.6
Mandible ($D_{\text{max}}$)	Gy	67.8±4.1	−0.3±1.4	−0.2±1.8	−0.2±1.6
Tyroid ($D_{\text{mean}}$)	Gy	46.8±8.8	0.0±0.4	0.3±0.6	0.4±0.6
Oral Cavity ($D_{\text{mean}}$)	Gy	47.8±10.5	−0.0±0.2	0.1±0.4	0.1±0.3
Constrictor Muscles ($D_{\text{mean}}$)	Gy	51.5±9.3	−0.1±0.3	0.1±0.3	−0.1±0.6
Larynx ($D_{\text{mean}}$)	Gy	44.6±7.3	−0.2±0.4	0.0±1.0	−0.0±1.1
${\text{CI}}_{\text{PTV}1}$	a.u.	0.89±0.04	0.01±0.01 ^a^	0.01±0.01 ^a^	−0.06±0.02 ^a^
${\text{CI}}_{\text{PTV}2}$	a.u.	0.65±0.03	0.01±0.01 ^a^	0.02±0.01 ^a^	−0.03±0.02 ^a^
Monitor Units	MU	959±111	9±34	69±61 ^a^	119±52 ^a^

a ^a^ Significant differences (p<0.05, by paired Wilcoxon test) of sparing plan (plans 2, 3, and 4) with respect to standard plan.

In the standard plan, the maximum doses to spinal cord and brain stem (43.5±2.2 Gy and 36.7±14.0 Gy respectively) satisfied commonly recommended values (maximum dose to spinal cord ≤45 Gy and maximum dose to brain steam ≤50 Gy), and the dose to their PRVs, obtained by isotropic 3 mm expansion, did not exceed 50 and 54 Gy, respectively. The maximum doses on PRV obtained by 5 mm expansion were 3‐4 Gy greater. The average maximum dose on the brain stem was smaller, since in some patients the position of the targets was distant from brain stem. In each patient, a marked significant reduction was obtained in the sparing plans. The average difference of maximum dose were around −16 Gy to spinal cord and around −10 Gy to brain stem in both marked‐sparing and low‐conformity plans, which provided comparable sparing. The average difference in moderate‐sparing plan was obviously smaller (around −7 and −4 Gy), due to the higher doses applied to the objectives.

In the standard plan, $\text{PTV}1_{95\%}$ and $\text{PTV}2_{95\%}$ were always >95%. In the moderate‐sparing plan, a small significant difference was observed in $\text{PTV}1_{95\%}$ (−0.5%±0.6%) and only very small difference in $\text{PTV}2_{95\%}$ (−0.2%±0.2%). Greater significant differences were observed in $\text{PTV}1_{95\%}$ (−1.2%±0.8%) and $\text{PTV}2_{95\%}$ (−0.6%±0.4%) in the marked‐sparing plan. In the low‐conformity plan, no significant difference was observed in $\text{PTV}1_{95\%}$ and only a very small difference in $\text{PTV}2_{95\%}$ (−0.3%±0.3% from the initial 98.3%±0.8% in the standard plan). In all plans, using an expansion of 3 mm to delineate the PTVs, the average $\text{PTV}1_{95\%}$ and $\text{PTV}2_{95\%}$ were around 99%.

In the low‐conformity plan, the improved target coverage was obtained at the expense of a significant decrease in both ${\text{CI}}_{\text{PTV}1}$ and ${\text{CI}}_{\text{PTV}2}$. Due to the different isodoses used for their computation (65.8 Gy and 53.3 Gy, respectively), ${\text{CI}}_{\text{PTV}1}$ was always greater than ${\text{CI}}_{\text{PTV}2}$ (e.g., 0. 89 ± 0.04 versus 0.65 ± 0.03 in standard plan).

The effect of varying spinal cord/brain stem and conformality objectives in the four plans is clearly shown in Fig. 1, where each patient values are reported. The dose sparing on spinal cord and brain stem produced a worsening on target coverage from standard to marked‐sparing plan, whereas in low‐conformity plan the PTV1 coverage returned similar to standard plan. In seven patients, the $\text{PTV}1_{95\%}$ was lower than 95% in marked‐sparing plan, but always >95% in low‐conformity plan. In each patient and in all plans the $\text{PTV}2_{95\%}$ was greater than 95%, and the relative differences between standard and low‐conformity plans were smaller than the interpatient differences. Figure 1 also shows the decreases in CI in each patient in low‐conformity plan.

The mean changes on all the other OARs were not significant (Table 3). Any small differences occurring in some patients was much smaller than interpatients variability (see, for example, the left parotid reported in Fig. 1), as also demonstrated be the standard deviations reported in Table 3. It was not always possible to satisfy the objective during optimization, as the values were often above the recommended values. Depending on the side of PTV1, in some patient the parotids were asymmetrically spared (Fig. 1).

The dose sparing from standard to low‐conformity plan produced an increase in plan complexity, as evidenced by the increase in MUs, which was significant on marked‐sparing and low‐conformity plans (Table 3). On average, the MU increase was around 10% in low‐conformity plan with respect to standard plan, but also in this case the interplans differences were much smaller than interpatients differences (Fig. 1).

**Figure 2:**
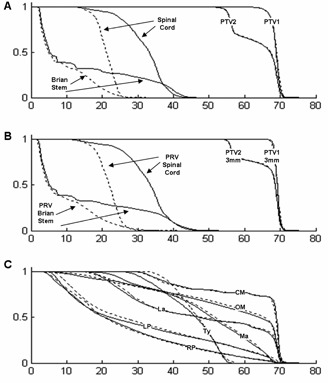
Dose‐volume histograms of patient #12, reporting the normalized cumulative doses of both the standard plans (continuous lines) and of the sparing low‐conformity plan (dashed lines). In the abscissa the dose is reported in Gy. (a) spinal cord, brain stem, PTV1 and PTV2; (b) ${\text{PRV}}_{3\text{mm}}$ of spinal cord, and $\text{PTV}1_{3\text{mm}}$ and $\text{PTV}2_{3\text{mm}}$ of brain stem; (c) left parotid (LP), right parotid (RP), mandible (Ma), thyroid (Ty), oral mucosa (OM), constrictor muscles (CM), larynx (La).

**Figure 3:**
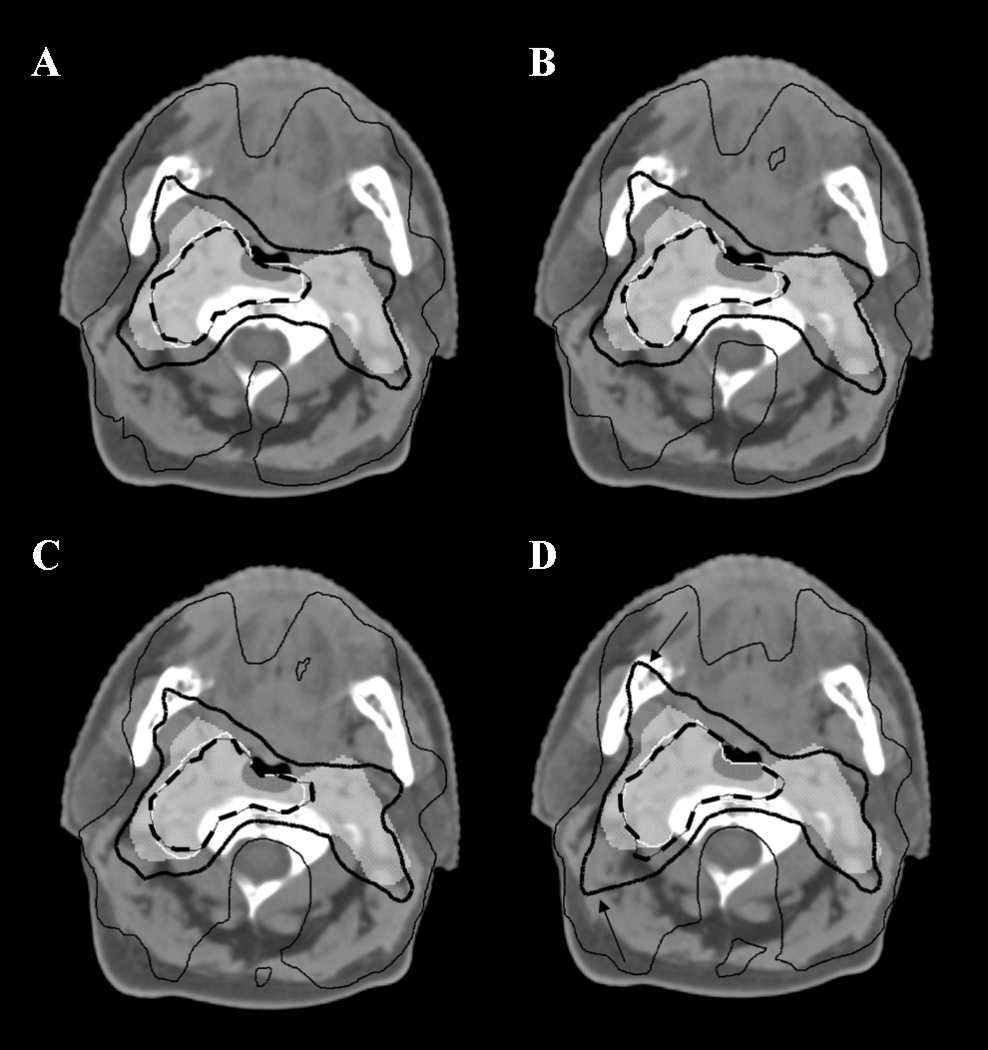
Dose distributions for patient #3: standard (a), moderate‐sparing (b), marked‐sparing (c), and low‐conformity (d) plans are displayed in the same axial slice. The white area represents PTV2 and the white line the PTV1 contour. Isodose lines are reported in black: dashed thick line=65.8 Gy (i.e., 95% of the dose prescribed to PTV1); continuous thick line=53.3 Gy (i.e., 95% of the dose prescribed to PTV2); continuous thin line=30 Gy. The black arrows show the enlargement of the 53.3 Gy isodose in low‐conformity plan.

As an example, the dose‐volume histograms of patient #12 are reported in Fig. 2. A clear marked reduction was visible in spinal cord and brain stem, whereas the differences in PTVs were small (Figs. 2(a)‐(b)). The corresponding changes in the other OARs were negligible (Fig. 2(c)). The isodoses on axial slices of patient #3 are reported in Fig. 3. The 30 Gy isodose clearly showed the sparing in marked‐sparing and low‐conformity plans with respect to standard plan. In agreement with the reduced CI, the 53.3 Gy isodose resulted enlarged in low‐conformity plan (Fig. 3(d)).

## IV. DISCUSSION

Many approaches have been tried in order to reduce the rate of locoregional relapse and improve the poor response in locally advanced head and neck cancer. Dose escalation to the target, reducing the field margin while keeping the dose to OARs unchanged, is one of the possible techniques.^27^, ^28^ Unfortunately, improved outcomes driven by dose intensification can be accompanied by an increase in acute and late toxicities, which can affect social and functional outcomes.^(29)^ Altered fractionation using hyperfractionated schemes is another approach. A large meta‐analysis of 15 randomized trials indicated that altered fractionation schedules increased overall survival and locoregional control in patients receiving definitive radiation alone.^(30)^ However, tumor control is gained at the expense of increased toxicity, which may in turn interfere with compliance and timely treatment delivery. It follows that, in the current standard of treatment, the rate of locoregional relapse could remain high, and reirradiation is candidate to become a common practice, at least in highly recurrent tumors.

Since the application of lower dose constraints to spinal cord and brain stem in the initial irradiation might facilitate reirradiation, it might result helpful in a considerable number of patients. The maximal dose on spinal cord and brain stem data reported in previous studies on the initial head and neck irradiation planned by IMRT are variable, depending also on the location and extension of the target volumes. The Report No. 83 of the International Commission on Radiation Units and Measurements (ICRU) recommended a maximum dose to spinal cord <48 Gy, but usually the doses reported in the literature were well below this value. In Widesott et al.,^(31)^ for example, the reported maximum doses to spinal cord were 36.5±0.6 Gy and 32.0±3.4 Gy, for tomotherapy and protontherapy, respectively.

In our study, a moderate difference (around −7 Gy) in the spinal cord was obtained from the initial values obtained in the standard plan (43.5±2.2 Gy), without compromising the sparing of the other OARS or dose conformity, and slightly affecting the coverage of PTVs. As expected, the maximum doses to PRVs were higher, particularly to ${\text{PRV}}_{5\text{mm}}$. However, the 3 mm margin was more consistent with the 5 mm margin of PTVs, considering that a smaller margin is usually applied to obtain the PRV with respect to PTV.^(32)^ Furthermore, the application of daily image‐guided can reduce the margin required to account for interfraction movements.^(33)^

However, such doses to spinal cord could still potentially limit reirradiation. Lower doses (e.g., <30 Gy) might be necessary to allow a more flexible reirradiation in recurrent patients, which might focus on reducing, as much as possible, the risk of developing the adverse effects not related to neuropathy (e.g., fistulae, carotid rupture, osteoradionecrosis, and soft tissue necrosis).

We explored the effect of a marked reduction in two different scenarios: compromising target coverage and compromising dose conformity. In the first scenario, the marked dose sparing on spinal cord and brain stem (around −16 and −10 Gy, respectively) was obtained at the expense of a small but significant worsening on dose distribution at the margin of the targets. Some caution should be used analyzing the difference on $\text{PTV}1_{95\%}$, at least in some patients. In fact, after initial irradiation by IMRT, recurrent and second primary tumors can arise in close proximity to previously irradiated fields. However, the majority of recurrences occur within high dose regions,^(34)^ and recent data demonstrate the safety of PTV reduction of less than 5 mm in the setting of daily image‐guided positioning.^(35)^ In our study, the coverage of the PTVs obtained by an expansion of 3 mm resulted very high and the reduction due to spinal cord and brain stem sparing resulted negligible. It can be concluded that only the coverage of the PTVs obtained by a 5 mm margin was affected by preventive sparing. In the second scenario, a similar marked sparing on spinal cord and brain stem was obtained accepting worse dose conformity, but without significant changes on $\text{PTV}1_{95\%}$ and with only negligible decrease in $\text{PTV}2_{95\%}$.

Considerably, in all the sparing plans, the dose reduction on spinal cord and brain stem were obtained without significant changes on the other OARs. The exploration of preventive sparing on other OARs (e.g., the parotids and the mandible) was beyond the purpose of the present study. The other OARs had major impact in limiting the dose to the PTVs, particularly when they partially overlapped with the PTVs. Accordingly, in our patients, the dose the other OARs were often above the recommended values, and usually they are already optimized to the minimum achievable dose in the initial irradiation.

The sparing procedure is not expected to affect treatment deliverability. The entire standard and the sparing plans were planned by DMPO using a fixed number of segments. It was previously reported that in head and neck treatment, the DMPO plans required fewer MU (around 42%) and fewer segments than conventional optimization methods,^(36)^ which translated directly into a decrease in treatment times. In our DMPO optimization, even the significant increase in MU we observed in the sparing plan (around 10%) was smaller than interpatient variability. Such increase was not expected to substantially improve plan complexity.

Finally, our results did not seem to depend on tumor site. Even if in most of the 18 consecutive patients the PTV1 was located in the oropharynx, a similar sparing was obtained also in the other sites. Despite this, such approach would be beneficial only in the patients with worse prognostic factors. Subgroup of patients has to be identified as best candidates, depending on tumor stage, grade, and site. As an example, considering that the results in carcinomas of the larynx and the hypopharynx using the most aggressive chemoradiation approaches remained poor, our results suggest that, in most of these patients, a preventive sparing should be recommended to make potential reirradiation safer.

## V. CONCLUSIONS

Radiotherapy for head and neck tumor recurrences or second primary cancers in irradiated tissue is an attractive therapeutic option. In order to improve the healthy tissues tolerance, a preventive sparing could be adopted by lowering OARs doses during the first course of irradiation. Our study demonstrated that, in head and neck IMRT, the typical suggested doses to spinal cord and brain steam may produce suboptimal dose distributions, since it is often possible to obtain a moderate, but significant, dose reduction in different tumor sites. A marked reduction can be obtained without worsening the dose to the other OARs, but at the expense of a small compromise on target coverage or at the expense of a decrease in dose conformity. The reported information can help radiation oncologists in their practice to select the better approach to make potential reirradiation safer.

Guidelines for initial irradiation should always recommend the preventive optimization to the minimum achievable levels. In particular, a preventive sparing should be recommended in locally advanced highly recurrent cancer, such as the carcinomas of the larynx and the hypopharynx, since in these patients there is a high probability of benefiting by successive reirradiation.

## References

[1] Vermorken JB and Specenier P . Optimal treatment for recurrent/metastatic head and neck cancer. Ann Oncol. 2010;21(Suppl 7):vii252–61. 20943624 10.1093/annonc/mdq453

[2] Begg AC . Predicting recurrence after radiotherapy in head and neck cancer. Semin Radiat Oncol. 2012;22(2):108–18. 22385918 10.1016/j.semradonc.2011.12.002

[3] Huang K , Xia P , Chuang C , et al. Intensity‐modulated chemoradiation for treatment of stage III and IV oropharyngeal carcinoma: the University of California, San Francisco experience. Cancer. 2008;113(3):497–507. 18521908 10.1002/cncr.23578

[4] de Arruda FF , Puri DR , Zhung J , et al. Intensity‐modulated radiation therapy for the treatment of oropharyngeal carcinoma: the Memorial Sloan‐Kettering Cancer Center experience. Int J Radiat Oncol Biol Phys. 2006;64(2):363–73. 15925451 10.1016/j.ijrobp.2005.03.006

[5] Lefebvre JL . Laryngeal preservation in head and neck cancer: multidisciplinary approach. Lancet Oncol. 2006;7(9):747–55. 16945770 10.1016/S1470-2045(06)70860-9

[6] Zbaren P , Weidner S , Thoeny HC . Laryngeal and hypopharyngeal carcinomas after (chemo)radiotherapy: a diagnostic dilemma. Curr Opin Otolaryngol Head Neck Surg. 2008;16(2):147–53. 18327034 10.1097/MOO.0b013e3282f702a9

[7] McDonald MW , Lawson J , Garg MK , et al. ACR appropriateness criteria retreatment of recurrent head and neck cancer after prior definitive radiation expert panel on radiation oncology‐head and neck cancer. Int J Radiat Oncol Biol Phys. 2011;80(5):1292–98. 21530100 10.1016/j.ijrobp.2011.02.014

[8] Ang KK , Harris J , Wheeler R , et al. Human papillomavirus and survival of patients with oropharyngeal cancer. N Engl J Med. 2010;363:24–35. 20530316 10.1056/NEJMoa0912217 PMC2943767

[9] Mouttet‐Audouard R , Gras L , Comet B , Lartigau E . Evidence based and new developments in re‐irradiation for recurrent or second primary head and neck cancers. Curr Opin Otolaryngol Head Neck Surg. 2012;20(2):137–41. 22249172 10.1097/MOO.0b013e3283506a52

[10] Janot F , de Raucourt D , Benhamou E , et al. Randomized trial of postoperative reirradiation combined with chemotherapy after salvage surgery compared with salvage surgery alone in head and neck carcinoma. J Clin Oncol. 2008;26(34):5518–23. 18936479 10.1200/JCO.2007.15.0102

[11] Zafereo ME , Hanasono MM , Rosenthal DI , et al. The role of salvage surgery in patients with recurrent squamous cell carcinoma of the oropharynx. Cancer. 2009;115(24):5723–33. 19760612 10.1002/cncr.24595

[12] Lee N , Chan K , Bekelman JE , et al. Salvage re‐irradiation for recurrent head and neck cancer. Int J Radiat Oncol Biol Phys. 2007;68(3):731–40. 17379449 10.1016/j.ijrobp.2006.12.055

[13] Wong SJ and Spencer S . Reirradiation and concurrent chemotherapy after salvage surgery: pay now or pay later. J Clin Oncol. 2008;26(34):5500–01. 18936468 10.1200/JCO.2008.19.0868

[14] Hoebers F , Heemsbergen W , Moor S , et al. Reirradiation for head‐and‐neck cancer: delicate balance between effectiveness and toxicity. Int J Radiat Oncol Biol Phys. 2011;81(3): e111–18. 21362581 10.1016/j.ijrobp.2011.01.004

[15] Sher DJ , Haddad RI , Norris CM Jr , et al. Efficacy and toxicity of reirradiation using intensity‐modulated radiotherapy for recurrent or second primary head and neck cancer. Cancer. 2010;116(20):4761–68. 20572036 10.1002/cncr.25305

[16] Sulman EP , Schwartz DL , Le TT , et al. IMRT reirradiation of head and neck cancer‐disease control and morbidity outcomes. Int J Radiat Oncol Biol Phys. 2009;73(2):399–409. 18556144 10.1016/j.ijrobp.2008.04.021

[17] Biagioli MC , Harvey M , Roman E , et al. Intensity‐modulated radiotherapy with concurrent chemotherapy for previously irradiated, recurrent head and neck cancer. Int J Radiat Oncol Biol Phys. 2007;69(4):1067–73. 17967302 10.1016/j.ijrobp.2007.04.057

[18] Chua DT , Sham JS , Leung LH , Au GK . Re‐irradiation of nasopharyngeal carcinoma with intensity‐modulated radiotherapy. Radiother Oncol. 2005;77(3):290–94. 16289398 10.1016/j.radonc.2005.10.010

[19] Chen AM , Farwell DG , Luu Q , Cheng S , Donald PJ , Purdy JA . Prospective trial of high‐dose reirradiation using daily image guidance with intensity‐modulated radiotherapy for recurrent and second primary head‐and‐neck cancer. Int J Radiat Oncol Biol Phys. 2011;80(3):669–76. 20547443 10.1016/j.ijrobp.2010.02.023

[20] Chen CC , Lee CC , Mah D , et al. Dose sparing of brainstem and spinal cord for re‐irradiating recurrent head and neck cancer with intensity‐modulated radiotherapy. Med Dosim. 2011;36(1):21–27. 20202815 10.1016/j.meddos.2009.10.005

[21] Schultheiss TE . The radiation dose‐response of the human spinal cord. Int J Radiat Oncol Biol Phys. 2008;71(5):1455–59. 18243570 10.1016/j.ijrobp.2007.11.075

[22] Kirkpatrick JP , van der Kogel AJ , Schultheiss TE . Radiation dose‐volume effects in the spinal cord. Int J Radiat Oncol Biol Phys. 2010;76(3 Suppl):S42–S49. 20171517 10.1016/j.ijrobp.2009.04.095

[23] Wu Q , Manning M , Schmidt‐Ullrich R , Mohan R . The potential for sparing of parotids and escalation of biologically effective dose with intensity‐modulated radiation treatments of head and neck cancers: a treatment design study. Int J Radiat Oncol Biol Phys. 2000;46(1):195–205. 10656393 10.1016/s0360-3016(99)00304-1

[24] Zwicker F , Hauswald H , Nill S , et al. New multileaf collimator with a leaf width of 5 mm improves plan quality compared to 10 mm in step‐and‐shoot IMRT of HNC using integrated boost procedure. Strahlenther Onkol. 2010;186(6):334–43. 20495969 10.1007/s00066-010-2103-8

[25] Jiang Z , Earl MA , Zhang GW , Yu CX , Shepard DM . An examination of the number of required apertures for step‐and‐shoot IMRT. Phys Med Biol. 2005;50(23):5653–63. 16306659 10.1088/0031-9155/50/23/017

[26] Zwicker F , Roeder F , Hauswald H , et al. Reirradiation with intensity‐modulated radiotherapy in recurrent head and neck cancer. Head Neck. 2011;33(12):1695–702. 21284054 10.1002/hed.21663

[27] Bhide S , Guerrero Urbano MT , Clark C , et al. Results of intensity modulated radiotherapy (IMRT) in laryngeal and hypopharyngeal cancer: a dose escalation study. Radiother Oncol. 2007;82:S74–S75.

[28] Guerrero Urbano T , Clark CH , Hansen VN , et al. A phase I study of dose‐escalated chemoradiation with accelerated intensity modulated radiotherapy in locally advanced head and neck cancer. Radiother Oncol. 2007;85(1):36–41. 17709149 10.1016/j.radonc.2007.07.011

[29] Murphy B . Advances in quality of life and symptom management for head and neck cancer patients. Curr Opin Oncol. 2009;21(3):242–47. 19322081 10.1097/CCO.0b013e32832a230c

[30] Bourhis J , Overgaard J , Audry H , et al. Hyperfractionated or accelerated radiotherapy in head and neck cancer: a meta‐analysis. Lancet. 2006;368(9538):843–54. 16950362 10.1016/S0140-6736(06)69121-6

[31] Widesott L , Pierelli A , Fiorino C , et al. Intensity‐modulated proton therapy versus helical tomotherapy in nasopharynx cancer: planning comparison and NTCP evaluation. Int J Radiat Oncol Biol Phys. 2008;72(2):589–96. 18793962 10.1016/j.ijrobp.2008.05.065

[32] Stroom JC and Heijmen BJ . Limitations of the planning organ at risk volume (PRV) concept. Int J Radiat Oncol Biol Phys. 2006;66(1):279–86. 16904527 10.1016/j.ijrobp.2006.05.009

[33] Duma MN , Kampfer S , Schuster T , et al. Do we need daily image‐guided radiotherapy by megavoltage computed tomography in head and neck helical tomotherapy? The actual delivered dose to the spinal cord. Int J Radiat Oncol Biol Phys. 2012;84(1):283–88. 22417803 10.1016/j.ijrobp.2011.10.073

[34] Shakam A , Scrimger R , Liu D , et al. Dose‐volume analysis of locoregional recurrences in head and neck IMRT, as determined by deformable registration: a prospective multi‐institutional trial. Radiother Oncol. 2011;99(2):101–07. 21621868 10.1016/j.radonc.2011.05.008

[35] Chen AM , Farwell DG , Luu Q , Donald PJ , Perks J , Purdy JA . Evaluation of the planning target volume in the treatment of head and neck cancer with intensity‐modulated radiotherapy: what is the appropriate expansion margin in the setting of daily image guidance? Int J Radiat Oncol Biol Phys. 2011;81(4):943–49. 20932680 10.1016/j.ijrobp.2010.07.017

[36] Jones S and Williams M . Clinical evaluation of direct aperture optimization when applied to head‐and‐neck IMRT. Med Dosim. 2008;33(1):86–92. 18262129 10.1016/j.meddos.2007.04.002

